# Cancer cell growth and survival as a system-level property sustained by enhanced glycolysis and mitochondrial metabolic remodeling

**DOI:** 10.3389/fphys.2012.00362

**Published:** 2012-09-12

**Authors:** Lilia Alberghina, Daniela Gaglio, Cecilia Gelfi, Rosa M. Moresco, Giancarlo Mauri, Paola Bertolazzi, Cristina Messa, Maria C. Gilardi, Ferdinando Chiaradonna, Marco Vanoni

**Affiliations:** ^1^SysBio Centre for Systems BiologyMilano and Rome, Italy; ^2^Department of Biotechnology and Biosciences, University of Milano-Bicocca, Piazza della ScienzaMilano, Italy; ^3^IBFM-CNR, Via Fratelli Cervi 93Segrate, Milano, Italy; ^4^Department of Health Sciences, University of Milano-BicoccaMilano, Italy; ^5^DiSCo, University of Milano-BicoccaViale Sarca, Milano, Italy; ^6^IASI-CNR Viale Manzoni 30Rome, Italy

**Keywords:** cancer research, systems biology, metabolism, mitochondrial dysfunction, emergent properties

## Abstract

Systems Biology holds that complex cellular functions are generated as system-level properties endowed with robustness, each involving large networks of molecular determinants, generally identified by “omics” analyses. In this paper we describe four basic cancer cell properties that can easily be investigated *in vitro*: enhanced proliferation, evasion from apoptosis, genomic instability, and inability to undergo oncogene-induced senescence. Focusing our analysis on a K-*ras* dependent transformation system, we show that enhanced proliferation and evasion from apoptosis are closely linked, and present findings that indicate how a large metabolic remodeling sustains the enhanced growth ability. Network analysis of transcriptional profiling gives the first indication on this remodeling, further supported by biochemical investigations and metabolic flux analysis (MFA). Enhanced glycolysis, down-regulation of TCA cycle, decoupling of glucose and glutamine utilization, with increased reductive carboxylation of glutamine, so to yield a sustained production of growth building blocks and glutathione, are the hallmarks of enhanced proliferation. Low glucose availability specifically induces cell death in K-*ras* transformed cells, while PKA activation reverts this effect, possibly through at least two mitochondrial targets. The central role of mitochondria in determining the two investigated cancer cell properties is finally discussed. Taken together the findings reported herein indicate that a system-level property is sustained by a cascade of interconnected biochemical pathways that behave differently in normal and in transformed cells.

## Introduction

High-throughput technologies (e.g., transcriptomics, proteomics, and metabolomics) aim to obtain a global molecular description of complex biological processes and to reach a deeper understanding of their behavior. The application of these technologies has characterized the last decade of biological research, called “the post-genomic era” and each post-genomic technique has already significantly contributed to molecular investigation of many aspects of physiological and pathological processes in many cellular types.

An important, widely analyzed target of post-genomic techniques is the very diversified cancer phenotype. Gene expression microarrays allowed to quantitatively characterize genome-wide transcriptional profiles of various cancer types (Rhodes et al., 2004; Shoemaker, 2006) showing that the expression of about one to two thousand genes may vary between each cancer type and its normal counterpart (Ruan et al., 2006; Kao et al., 2009) observing also a great variability of gene expression during cancer progression (Greaves and Maley, 2012). Statistical methods allowed to recognize specific gene expression “signatures” that are up or down regulated in cancer cells as compared to their normal counterparts (van de Vijver et al., 2002; Rhodes et al., 2004; Balestrieri et al., 2012).

Unfortunately these “signatures” have only a statistical value: for instance “signatures” of gene expression obtained from primary breast cancer that are found associated with recurrence of the disease are not able to predict the outcome for individual patients (Weinberg, 2007). This inability is linked to the fact that the identification of these signatures has not increased understanding of the molecular mechanisms at the basis of oncogenic transformation (Mata et al., 2005; Joyce and Palsson, 2006).

Considering that a large number of post-transcriptional modifications, relevant for cell function, are not captured by this type of analysis, interest has been directed toward proteomic techniques. Although this technology allowed at the beginning to detect only the more abundant proteins, it has been possible to recognize proteins specifically expressed in transformed cells and to propose the use of some of them as cancer biomarkers (von Eggeling et al., 2000; Russo et al., 2003; Stevens et al., 2004). Two issues are confronting proteomic technologies: the detection of proteins expressed in low amount and quantitative identification of proteins post-translationally modified at specific residues by phosphorylation, acetylation etc. Recent developments have allowed to improve the sensitivity of the technology so to estimate, for instance, that about 10,000 different types of proteins are expressed in a human cell, that contains a total of about a billion proteins (Beck et al., 2011; Nagaraj et al., 2011). Some structural proteins (of cytoskeleton, of ribosomes, of proteasome) and some catalytic proteins (of carbohydrate metabolism) are very largely expressed, while transcription factors and protein kinases are expressed in much lower amounts, with about 600 proteins covering 75% of cell mass (Nagaraj et al., 2011). Besides, cellular functions depend upon protein activity, which can be modulated not only by protein phosphorylation or acetylation, but also by allosteric regulation and cellular localization. This awareness has promoted the development of another high-throughput technology, metabolomics.

Since metabolism is the outcome of the overall regulation from genetic control to modulated kinetic activity of enzymes (Spratlin et al., 2009), it potentially offers a more faithful readout of cellular function (ter Kuile and Westerhoff, 2001; Griffin and Shockcor, 2004), that responds quickly to environmental changes. Metabolic profiling—comparing normal and transformed cells—has been used to identify new biomarkers (Chung et al., 2003) and to support cancer diagnosis (Boren et al., 2001; Serkova and Boros, 2005).

The real challenge posed by more and more efficient high—throughput technologies becomes the availability of computational methods able to extract information and to transform information into knowledge, so to define quantitative integrated rules able to increase understanding and to confer predictive ability.

## From “Omics” data to networks and beyond

A first step along this line is for instance the identification of differentially expressed genes or proteins, their hierarchical clustering and pathway analysis (Okabe et al., 2001; LaTulippe et al., 2002; Varambally et al., 2005; Wang et al., 2005). Quite often differentially expressed gene products are mapped onto protein–protein interaction (PPI) maps, so to give them a network structure, amenable to statistical and topological investigation, whose results offer a first idea of structure of the PPI map underlying a given function.

While network analysis offers the possibility to recognize the presence of motifs or to investigate network architecture and may suggest new lines of investigation, it does not substantially increase understanding on the molecular mechanism of complex cellular functions: at a minimum it needs to be accompanied by a different type of representation able to structure the network according to recognizable biochemical functions (Kaizu et al., 2010). Even sophisticated analyses that investigate the bistability of large-scale networks for various human cancers (Cui, 2010; Shiraishi et al., 2010), seem unable to provide the breakthrough that is required to extract a deeper understanding on cancer growth from post-genomic findings.

It is clear, at this point, that a change of paradigm is required since it is by now widely recognized that complex cellular functions are generated by the dynamic interactions of a large number of molecular components (DNA, RNA, proteins and small molecules) modulated by internal and external cues (Hartwell et al., 1999; Lauffenburger, 2000). Thus the function is not determined by a single component or a single level of organization, but is found distributed as an emergent (or system-level) property over many levels and components (Hartwell et al., 1999; Palumbo et al., 2010). Accordingly, a cell can be viewed as a system composed of many interconnected modules coordinately performing specific biological functions (metabolism, signaling, transcription, growth, cycle, autophagy, apoptosis, differentiation, etc.), each module being characterized by a network of interacting molecules (Hartwell et al., 1999; Eisenberg et al., 2000; Nurse and Hayles, 2011). These modules can conveniently be described according to the “circuit” metaphor, that is widely used by electronic engineers (Parhami, 2005) and is able to account for biological functions as system-level properties, in a more efficient and unambiguous way than network representation (Palumbo et al., 2010).

In this perspective, one has to determine the *structure* (i.e., the topology) and the *dynamics* (i.e., the behavior as a function of time) of the circuit. The analysis of circuit behavior in biological systems is quite well established from providing insight into signaling pathways (Bhalla and Iyengar, 1999) to investigate physiological angiogenesis (Niemisto et al., 2005). By using this approach a better understanding of the cell cycle in budding yeast (Alberghina et al., 2001, 2009, 2012; Barberis et al., 2007; Brummer et al., 2010; Palumbo et al., 2010) as well as in mouse fibroblasts (Alfieri et al., 2009) has been reached, allowing to identify two cell cycle regulatory functions as system-level properties: the requirement of a critical cell size to enter into S phase (Barberis et al., 2007) and the synchronous timing of the onset of DNA replication (Brummer et al., 2010; Salazar et al., 2011).

Since “Systems Biology deals with the mechanisms by which macromolecules produce the functional properties of living cells through dynamic interactions,” it follows that systems biology is needed to reach a more satisfying understanding of the relations between genotype and phenotype in cancer. The integration of molecular analysis with mathematical modeling and simulation, in an iterative process, characterizes the systems biology approach (Kitano, 2002; Alberghina and Westerhoff, 2005). Mathematical models may be constructed at different levels of resolution (Noble, 2002) and then simulation analysis will allow to detect system-level properties (Likic et al., 2010). The identification of the network of a complex function is often the first step for the construction of the corresponding mathematical model, offering the constraints for the interactions of the various partners, but it has to be complemented with more information on the spatio-temporal coordinates of the process and on the function obtained from cell physiology studies.

## A new systems biology approach to cancer

Many Authors have already applied mathematical modeling to cancer in a systems biology perspective (Khalil and Hill, 2005; Kreeger and Lauffenburger, 2010). Of great interest is the line of research of Westerhoff and collaborators that over several years have developed an innovative approach that takes into consideration specific modules of cancer phenotype, from the activation of signaling (Hornberg et al., 2006), to metabolism (Moreno-Sanchez et al., 2010), at the same time offering tools to integrate various modules and levels of organization (Hornberg et al., 2006; Bruggeman et al., 2008).

In the following we are going to present our approach to the problem that has many elements of novelty, while it follows Westerhoff's line of thought. We start from the consideration, made by Hanahan and Weinberg (2000, 2011), that despite a great cellular and molecular variability in cancer phenotypes, it is possible to identify a restricted number of physiological alterations which together define the phenotype of most human malignancies. They include: unlimited proliferation potential, self-sufficiency in growth signals, resistance to anti-proliferative and pro-apoptotic cues, sustained angiogenesis, ability to metastasize to distinct organs, reprogramming of cellular energy metabolism, evasion of cancer cells from attack and elimination by the immune system, genomic instability, tumor-promoting inflammation, presence of a stress phenotype, that although not required for initiating tumorigenesis is apparently required for tumor maintenance (Hanahan and Weinberg, 2011). Some of these properties can be analyzed at the cellular level, while others are executed at the organismal level.

We focus our attention on cellular hallmarks that may be easily analyzed *in vitro*, given that they are relevant to sustain organismal features. We reorganize in a different way more amenable to experimental analysis some classical cancer hallmarks: for example we consider *enhanced cell proliferation* that includes unlimited proliferation, independence from growth factors and reprogramming of energy metabolism. Following this line of thought, we came to recognize four basic cancer properties: *enhanced cell proliferation*, *evasion from apoptosis*, *genomic instability*, *inability of cancer cells to enter senescence*, each considered as a system-level property (Bhalla and Iyengar, 1999; Chiaradonna et al., 2012), able to be disassembled in modules, that can be as small as a single multi-domain protein (Sacco et al., 2012) or a protein complex.

The strategy that we follow and that—as outlined in section From “Omics” Data to Networks and Beyond” above—has been employed with success to investigate yeast growth and cycle can be summarized as follows: to use genome-wide analysis and molecular-tailored experiments to ascertain which molecular components are involved in the function under evaluation; to take into consideration all pertinent literature and make use of chemical and/or genetic perturbations to reconstruct the sequence of molecular events that underlay the function under analysis; to design a concept map of the function describing also relevant spatio-temporal coordinates; use the concept map to construct a mathematical model, in terms of chemical kinetics; to estimate, both from literature and from *ad hoc* experiments, relevant parameters of the model (concentration of molecular species; binding constants, etc.) as well as quantitative estimation of the system-level property, under various experimental conditions; to test the adequacy of the model by simulation of system's behavior under a variety of conditions; to run sensitivity analysis to assess the relevance of each element of the model in determining the system-level property.

Of course, as said before, a model can be constructed at various levels, considering different molecular players, as shown for instance in the case of the circuit “osmostat” in the homeostatic response to hyperosmotic stress (Klipp et al., 2005; Gennemark et al., 2006; Schaber et al., 2011), with each model able to put in evidence relationships that are obscured in other versions.

## Two basic system-level properties of cancer cells

The more obvious phenotype of cancer cells is given by their uncontrolled proliferation, due to an enhanced cellular growth, insensitive to anti-proliferative signals, and to a strong reduction of their response to pro-apoptotic cues.

Among the various activated signaling pathways involved in cellular transformation, a relevant place has to be given to *ras*, whose oncogenic mutations are found in a large number of human tumors (Bos, 1989; Rodenhuis, 1992). The relevance of oncogenic *ras* mutations in different malignant phenotypes derives from the fact that Ras proteins are intracellular switches whose activation state—i.e., their binding to GDP and GTP—controls downstream pathways leading to cell growth and differentiation. Their activation state depends on the competing action of GTPase Activating Proteins (GAPs) and Guanine nucleotide Exchange Factors (GEFs). Deregulation of either GAP or GEF activity may result in hypo- or hyper-activation of downstream pathway(s), so that for instance over-expression of a GEF or inactivation a GAP may both result in cell transformation (Reuther and Der, 2000; Downward, 2003; Konstantinopoulos et al., 2007). Different Authors have shown that attenuation of oncogenic Ras signaling in cancer cells, by using Ras or GEF dominant negative proteins, reverts *ras*-dependent cancer cells to normal phenotype on the basis of morphology, anchorage-independent growth, ability to proliferate and strong reduction of tumor formation in a nude mice model (Vanoni et al., 1999; Bossu et al., 2000; Stewart and Guan, 2000; Fiordalisi et al., 2002; Oliva et al., 2004; Ford et al., 2009). Therefore, cancer proliferation shows addiction to K-*ras*.

As discussed in a recent review (Pylayeva-Gupta et al., 2011), oncogenic *ras* activation promotes the stimulation of several signaling pathway (Raf-MAPK, PI3K, Rho-Rac, and Ral-Gef) that converge to activate cyclin D1 synthesis and stabilization, whose increase together with the down-regulation of cyclin-dependent kinase inhibitors, p27 and p21 promotes entrance into S phase (Rivard et al., 1999; Sa and Stacey, 2004).

The strong mitogenic stimulation imposed by K-*ras* activates DNA replication and hence the DNA damage response (DDR) (Bartkova et al., 2005; Gorgoulis et al., 2005; Di Micco et al., 2006; Koorstra et al., 2009). In normal cells, that possess a functional DNA damage checkpoint machinery, DDR stimulation by oncogenic *ras* brings to cell cycle arrest in a process called oncogene-induced-senescence (OIS) (Serrano et al., 1997; Ferbeyre et al., 2002; Di Micco et al., 2006). In transformed cells (in which other mutations, for instance loss of p53, are present) genomic instability is induced, that is going to have a relevant role in the clonal evolution of cancer cells (Loeb and Loeb, 2000; Little, 2010).

Beside its role in stimulating cell growth, a relevant action of oncogenic *ras* has been reported in suppression of apoptosis, a complex process that can be activated by extracellular cues such as withdrawal of growth factor or detachment from the matrix, or it may be triggered by intracellular events such as DNA damage or mitochondrial dysfunction. The extrinsic and the intrinsic pathways converge in the activation of caspase-3 and they are regulated by the fine balance of network of pro-apototic and anti-apototic molecules that respond to a large number of cellular cues (Liou et al., 2000; Cox and Der, 2003; Fritz and Fajas, 2010). The anti-apoptotic role of oncogenic *ras* involves both the increase of levels of anti-apoptotic proteins—such as BAK and IAPs—as well as the inactivation of pro-apototic proteins, such as BAD which is inactivated by phosphorylation (Gewies, 2003).

One of the standard procedures in systems biology consists in perturbing the system by chemical or genetic alterations and monitoring its behavior (Alberghina and Westerhoff, 2005): by changing nutrient availability, differential growth and apoptotic responses have been shown to be elicited in normal and cancer cells.

An initial concentration of 25 mM glucose (HG) and of 4 mM glutamine (HQ) is provided to cells growing under optimal condition, while glucose limitation is imposed by an initial concentration of 1 mM (LG) and glutamine limitation by one of 0.5 mM (LQ). As previously described, *ras* activation induces OIS in normal cells (Serrano et al., 1997; Ferbeyre et al., 2002; Di Micco et al., 2006), while in transformed cells it promotes sustained cell growth and evasion from senescence and apoptosis (Serrano et al., 1997; Ferbeyre et al., 2002). Glucose limitation induces G_1_ arrest both in normal and in transformed cells, but it specifically enhances cell death in transformed cells (Chiaradonna et al., 2006). Glutamine limitation induces G_1_ arrest in normal cells while transformed cells do not arrest in G_1_, but make an abortive entrance into S phase, which is partially relieved by supplying ribonucleotides in the medium (Gaglio et al., 2009).

By comparing transformed to normal cells, in the next sections we analyze the molecular players of the very large module “enhanced cell growth” as well as those of the two connected modules “evasion from apoptosis” and “LG-induced apoptosis” considering both findings from our laboratory and literature data.

### “Enhanced cell growth”

Both *in vivo* and *in vitro*, normal cells, when they reach the correct dimension of the organ to which they belong or the critical cell density—confluence—respectively, stop growing upon *contact inhibition* (Eagle and Levine, 1967; Holley and Kiernan, 1968). In similar conditions, transformed cells continue to proliferate and hence show an “enhanced cell growth” (Abercrombie, 1979; Fagotto and Gumbiner, 1996). Therefore, cancer cells rather than an accelerated proliferation show an ability to avoid contact inhibition. In fact when grown *in vitro* both normal and transformed cells show the same initial growth rate when nutrients and factors availability is not limiting (Chiaradonna et al., 2006).

Several Authors have tried to enlighten the molecular mechanisms of contact inhibition. A recent report (Kuppers et al., 2010) identified a large set of differentially expressed genes in contact-inhibited as compared to sparsely growing NIH3T3 cells. The majority of these genes was up-regulated suggesting that contact-inhibition is an actively induced state. A sizable part of up-regulated genes appears linked to general cellular metabolic processes such as glutathione and nucleotide synthesis. Sustained metabolic activity in contact-inhibited fibroblasts has been independently confirmed in primary human fibroblasts using flux analysis techniques (Lemons et al., 2010). Transcriptional profiling indicates that in transformed cells high cell density does not activate the metabolic response typical of contact inhibition (Chiaradonna et al., 2012).

As shown since the 1920s by Otto Warburg, cancer cells are characterized by an enhanced utilization of glucose by aerobic glycolysis with a reduction of mitochondrial oxidative phosphorylation, the so called Warburg effect (Warburg et al., 1927). Transcription of many genes encoding glycolytic enzymes is up-regulated in transformed NIH3T3 fibroblsts harboring a K-*ras* gene mutated in codon 12 (Chiaradonna et al., 2006). Morphological, biochemical, and genetic mitochondrial dysfunction has been reported to occur in several cancer cells (Modica-Napolitano and Singh, 2004; Shidara et al., 2005; Wallace, 2005). In K-*ras* transformed cells a substantial down-regulation of oxidative phosphorylation, accompanied by a strong reduction of Complex I content and activity, has been reported together with an increase of mitochondrial membrane potential and a reduction of the NADH ubiquinone reductase activity (Baracca et al., 2010).

The decrease of ATP forming ability of cancer mitochondria is compensated by the ATP produced by glycolysis because, in the time required by a cancer cell in normoxic condition to completely oxidize one molecule of glucose through the TCA cycle so to produce 36 molecules of ATP, ten more molecules of glucose are converted to lactic acid to make additional 20 molecules of ATP (Koppenol et al., 2011). Thus in the time period in which a normal cell produces 36 ATP from a molecule of glucose, a cancer cell appears able to produce 56 of them. Of course these calculations are only indicative, but they suggest that the very large and fast flux of glycolysis—as compared to the slow rate of the TCA cycle—may mediate a large pyruvate utilization that sustains the energy requirement for cancer cell growth.

A very large number of reports reviewed in (Koppenol et al., 2011) have investigated the role of mitochondrial mutations, of tumor suppressor genes and oncogenes in sustaining cellular transformation, showing that in all cases there is an activated signaling pathway followed by deregulation of glycolysis.

At this stage it is possible to conclude that the enhanced growth of cancer cells is accompanied by a profound rewiring of glycolytic and mitochondrial metabolism. Whether this rewired metabolism is the force driving cancer growth will be discussed later on.

In order to evaluate whether the mouse fibroblast model of K-*ras*-induced transformation, used in many of the characterization experiments of enhanced cell growth discussed so far, can be considered a representative model of molecular events taking place in human tumors, we run a comparative gene expression profile analysis (Balestrieri et al., 2012) over a set of human cancer cell lines (NCI-60 cell collection). In both species the transformation process involves a change in transcriptome (mostly by activation) being the transcriptional response of the mouse model significantly close to that of K*-ras*-dependent human tumors. A set of about 350 modulated genes is common to both mouse and human transformed cells, the processes more profoundly affected by transformation being: cellular metabolism, molecular biosynthesis, oxidative phosphorylation with significant alterations of mitochondrial structure and function. These findings support the notion that K-*ras* transformed mouse fibroblasts are a valid model of human cancer. Therefore we moved to analyze conditions that affect apoptosis in these cells.

### Evasion from apoptosis and low glucose-induced apoptosis: role of PKA pathway

We previously proposed that resistance of cancer cells to apoptosis may be linked to their alterations in mitochondria structure and function (Chiaradonna et al., 2012) being for instance interesting the observation that cancer cells have small mitochondria (Baracca et al., 2010) with a prevalence of fission over fusion (Palorini et al., 2012), that large mitochondria are resistant to autophagy (Galluzzi et al., 2011) and that autophagic vesicles are detectable in death-stressed cells undergoing apoptosis (Codogno and Meijer, 2005; Fulda et al., 2010).

Recent reports provide interesting insight on the biochemical pathways that connect glucose starvation to specific apoptosis activation in cancer cells. One of the pathways linking nutrient availability to metabolic and energy activities of the cell is the cAMP/PKA pathway (De Rasmo et al., 2011; Palorini et al., 2012). It stimulates glucose transport and utilization (Hiraki et al., 1989; Hosaka et al., 1992; Osawa et al., 1995), regulates mitochondrial dynamics (Chang and Blackstone, 2007; Cribbs and Strack, 2007), respiratory activity (Papa, 2006; Papa et al., 2008; De Rasmo et al., 2011) and apoptosis (Harada et al., 1999; Bellis et al., 2009). cAMP is compartmentalized in cytosol and in mitochondria (Zippin et al., 2003; Sardanelli et al., 2006; De Rasmo et al., 2009) with an increase of cytosolic cAMP able to enhance the activity of Complex I (NADH-ubiquinone oxidoreductase) and to decrease formation of reactive oxygen species (ROS) (Piccoli et al., 2006; Papa et al., 2008).

K*-ras*-dependent human tumor cell lines of the NCI-60 collection (Shoemaker, 2006) have been analyzed by transcriptional profiling. Statistical analysis and hierarchical clustering of PKA-related genes have been performed to show that in cancer cells there is down-regulation of the large part of the transcripts of the cAMP/PKA pathway (Balestrieri et al., 2009), thereby indicating that K-*ras* dependent oncogenic transformation may involve reduction of cAMP/PKA pathway activity.

Exogenous stimulation of PKA activity, obtained by treatment with the adenylate cyclase activator forskolin (FSK) (Hedin and Rosberg, 1983) protects K-*ras* transformed mouse and human cells from apoptosis induced by glucose shortage resulting in increased Complex I activity, ATP production, prevalence of fusion over fission and decrease in ROS production (Palorini et al., 2012). Reduction of the functional activity of PKA, observed in transformed cells, is not due to changes in the content of the enzyme, but derives from its altered responsiveness to regulatory molecules. Chemical inhibition of PKA activity reversed most of the effects induced by FSK (Palorini et al., 2012).

Phosphorylation of mitochondrial proteins has been reported by many Authors (Technikova-Dobrova et al., 2001; Chang and Blackstone, 2007; Acin-Perez et al., 2009). Two targets of the PKA promotion of cell survival in LG may be suggesyed. The NDUFS4 subunit of Complex I is critically relevant for Complex I activity. As recently discussed (Papa et al., 2011), activated cytosolic PKA phosphorylates NDUFS4, promoting its mitochondrial import. Once arrived in the matrix, NDUFS4—that is critically relevant for Complex I activity—may stabilize Complex I in its active form, being known that Complex I dysfunction generates ROS production. NDUFS4 localizes in the Complex I domain facing the matrix, thereby specifically exposing the protein to oxidative damage by ROS. Activation of the cAMP/PKA pathway may thus promoting the import of newly synthesized NDUFS4 protein which in a dynamic exchange with ROS-inactivated subunit may maintain Complex I activity (Papa et al., 2011).

The Drp1 protein, which promotes mitochondrial fission is inactivated by PKA phosphorylation (Dagda et al., 2011). Treatment with FSK of K-*ras* transformed human cells (MDA-MB-231) promotes Drp1 phosphorylation, increases mitochondrial fusion and reduces ROS production (Palorini et al., 2012). Besides, a link between respiratory chain activity (in particularly that of Complex I) and mitochondrial fusion/fission has recently been reported (Koopman et al., 2005; Benard et al., 2007).

The tentative conclusion that can be drawn at this point is that only small mitochondria with dysfunctional Complex I, reduced respiratory activity and active ROS production are susceptible to LG-induced apoptosis. When these features are reversed by PKA activation even in cancer cells (harboring other mutations besides activated K-*ras*) mitochondria are no longer susceptible to apoptosis promoted by glucose deprivation. It is interesting to recall at this point that a myc-inducible human Burkitt lymphoma cell line does not show LG-induced apoptosis, its survival being sustained by glutamine metabolism (Le et al., 2012).

Therefore, mitochondria appear to have a central role in two of the more basic system-level functions of cancer cells: enhanced growth and glucose-shortage-induced apoptosis. In the following a detailed analysis of mitochondrial metabolism in cancer cells is presented.

## Mitochondrial metabolism remodeling in cancer cells

Several reports have demonstrated the importance of metabolic changes induced by oncogenic K-*ras* in the onset of transformed phenotype, as reviewed in Drosten et al. (2010). In addition to its role in the activation of glycolysis in cancer cells (Ramanathan et al., 2005; Drosten et al., 2010), K-*ras* induces *de novo* lipid synthesis (Fritz and Fajas, 2010). Moreover, Weinberg et al., recently reported that the major function of glycolytically-produced ATP is the energetic support of growth under hypoxic conditions and showed that glutamine conversion into α-ketoglutarate (AKG) is essential for K-*ras*-induced anchorage-independent growth (Weinberg et al., 2010).

Metabolic flux analysis (MFA, described in greater detail in “Studying the Dynamics of Metabolic Network”) is a recent development of metabolomic techniques. By combining the use of isotopic tracers and computer algorithms, MFA allows to actually estimate in quantitative terms intracellular fluxes along metabolic pathways (Metallo et al., 2009; Hiller et al., 2010), is going beyond standard metabolomics that generates “snapshots” of metabolic profiles in different experimental conditions.

**Figure 1:**
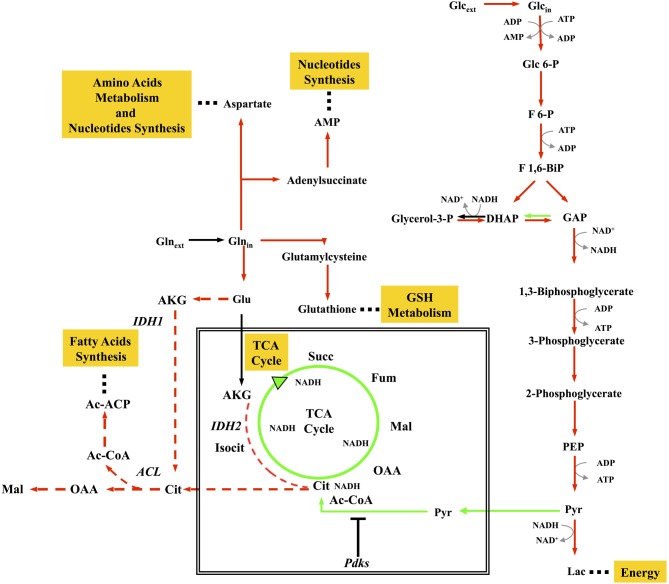
**Concept map of metabolic remodelling in cancer cells.** The map shows some of the more important aspects of metabolism in proliferating cancer cells, including enhanced glycolysis; downregulation of TCA cycle; stimulation of reductive carboxylation of glutamine sustaining biosynthesis of proteins, nucleotides, and lipids. Red arrows represent enhanced flux, green arrows represent reduced flux, black arrows represent unchanged flux, red dotted arrows represent enhanced reductive carboxylation flux.

By combining MFA and transcriptional profiling (Gaglio et al., 2011), we could show that glucose and glutamine pathways are decoupled in K-*ras* transformed cells. In both murine and human colon carcinoma HCT116 cells, oxidation of pyruvate to acetyl Co-A is substantially decreased, probably due to inhibition of pyruvate dehydrogenase (PDH) by pyruvate dehydrogenase kinase largely expressed in these an other cancer cells (Roche and Hiromasa, 2007). This leads to a reduced flux of glucose-derived carbon through the TCA cycle (Figure 1). Glucose is converted to lactate to produce ATP and at the same time glutamine provides both carbon and nitrogen for the synthesis of building blocks (amino acids, nucleotides) and glutathione through amino acyl transferase (AAT) and glutamate dehydrogenase (GLUD) activities (Figure 1). Building blocks and glutathione production, sustaining growth and the ability to quench ROS production, promote cancer cell proliferation and survival. Global gene expression analyses of different isogenic HCT116-derived colon carcinoma lines showed that disruption of both HIF-1α and HIF-2α or oncogenic activation of the K-*ras* decreased aerobic respiration and ATP production, with increased ROS generation (Chun et al., 2010). Consistent results have also been obtained by proteomics (Kang et al., 2012).

These data are in complete agreement with the large literature on cancer cell metabolism (DeBerardinis and Cheng, 2010; Metallo et al., 2011; Anastasiou and Cantley, 2012; Le et al., 2012; Ying et al., 2012). An important role of glutamine in determining cell fate was previously demonstrated for K-*ras* transformed cells (Gaglio et al., 2009), as well as for cells transformed by *myc* oncogene (Yuneva et al., 2007; Yuneva, 2008). Several studies have also reported the role of tumor suppressors, such as p53 and PTEN in the control of glycolytic and oxidative metabolism (Lian et al., 2006; Matoba et al., 2006). More recently, p53 has been assigned an important regulatory role in glutamine metabolism and in maintaining the balance between glycolysis and oxidative phosphorylation (Hu et al., 2010; Suzuki et al., 2010; Vousden, 2010), leading Wise and Thompson to propose that “glutamine addiction” could offer a new therapeutic target for cancer (Wise and Thompson, 2010). Since then, several other reports along the same line have been published, mostly dealing with the effect of the *myc* oncogene on glutamine utilization. Using MFA, Le et al. (2012) could show that under glucose deprivation, an alternative, energy-generating glutaminolysis pathway involving a glucose-independent TCA cycle is operative in a *myc*-inducible human Burkitt lymphoma cell line. In other cancer cells—including the K-*ras* transformed fibroblasts described herein—glutamine alone is unable to sustain growth and glucose deprivation induces cell death. Accordingly, inhibition of expression of the gene encoding lactate dehydrogenase A (LDHA) by siRNA or LDHA inhibition by a small-molecule inhibitor induces significant cell death and inhibits progression of human lymphoma and pancreatic cancer xenografts (Le et al., 2010). LDH-A silencing has also been reported to significantly decrease tumorigenicity ability of breast cancer cells (Wang et al., 2011). Besides acting as a nitrogen source for nucleotide and amino acid biosynthesis, glutamine plays a signaling role in TOR activation (Wise and Thompson, 2010).

AKG derived from glutamate is converted to isocitrate by isocitrate dehydrogenase 2 (IDH2), possibly relieving the burden on NADH reoxidation given by the dysfunction of Complex I, previously discussed to be a relevant characteristic of K-*ras*-dependent cancer cells. Recently, Metallo and colleagues, have shown in a variety of human cancer cell lines, both under hypoxic and normoxic conditions, that reductive metabolism of AKG promotes lipid biosynthesis (Figure 1) (Metallo et al., 2011). Similar results have been obtained by Wise and colleagues, that have shown an increased glutamine-dependent citrate production in hypoxic cells from reverse flux of AKG by mitochondrial IDH2 activity (Wise et al., 2011).

Besides, cancer-associated mutations in the IDH1-encoding gene result in a new ability of the enzyme to catalyse the NADPH-dependent reduction of AKG to R(–)-2-hydroxyglutarate (2HG) (Dang et al., 2009). 2HG has then been identified as an “oncometabolite” in a subset of gliomas harboring mutations in the genes encoding IDH1 and 2 (Kalinina et al., 2012). 2HG-producing IDH mutants are impaired in histone demethylation that is required for lineage-specific terminal differentiation of progenitor cells (Lu et al., 2012). Mutations in the IDH1-encoding gene are sufficient to establish the glioma hypermethylator phenotype, a powerful determinant of glioma pathogenicity (Turcan et al., 2012). (R)-2HG, but not (S)-2HG, stimulates prolyl 4-hydroxylases, thereby reducing HIF levels, event that promotes transformation as judged by enhanced proliferation and soft agar growth of human astrocytes (Koivunen et al., 2012).

In some cancer cells, a large amount of glycolytic carbon is diverted to serine and glycine through phosphoglycerate dehydrogenase (PHGDH) (Locasale et al., 2011). The *PHGDH* gene is found amplified in several tumors, while ectopic expression of PHGDH predispose to tumorigenesis and inhibition of PHGHDH expression reduces proliferation of cancer cell lines (Locasale et al., 2011). Besides, sarcosine, an N-methyl derivative of glycine has been identified non-invasively in urine as a differential metabolite that is largely increased during prostate cancer progression (Cavaliere et al., 2011; Wu et al., 2011). High resolution mass spectrometry (MS) on isolated mitochondria has been recently used to provide global metabolic information (Roede et al., 2012), an approach that could be extended to obtain a fine characterization of the differences between normal and cancer cells.

Taken together these findings indicate that there is an extensive metabolic remodeling in cancer cells in both cytoplasm and mitochondria and that these metabolic changes may deeply affect the interplay between genetic and epigenetic changes in human cancers. Cancer-associated alterations in metabolism should no longer be regarded as an indirect response to cell proliferation and survival signals (Ward and Thompson, 2012). It is becoming increasingly clear that cellular signaling and metabolism are deeply intertwined in a “two-way street” (Wellen and Thompson, 2012) allowing fine tuning of metabolism, cell death/survival and proliferation.

## Mitochondria as a focus for a systems biology approach to cancer

Both “enhanced growth” and “evasion from apoptosis/LG-induced apoptosis”, i.e., relevant system-level properties of cancer cell proliferation, largely rely on mitochondrial remodeling. For this reason, it is indispensable, in order to develop a sound systems biology approach, to utilize several convergent techniques able to yield a detailed molecular picture of the changes induced in mitochondria by the passage from normal to cancer cell proliferation: from bioenergetics analysis to metabolomic profiling, from proteomics to bioimaging.

**Figure 2:**
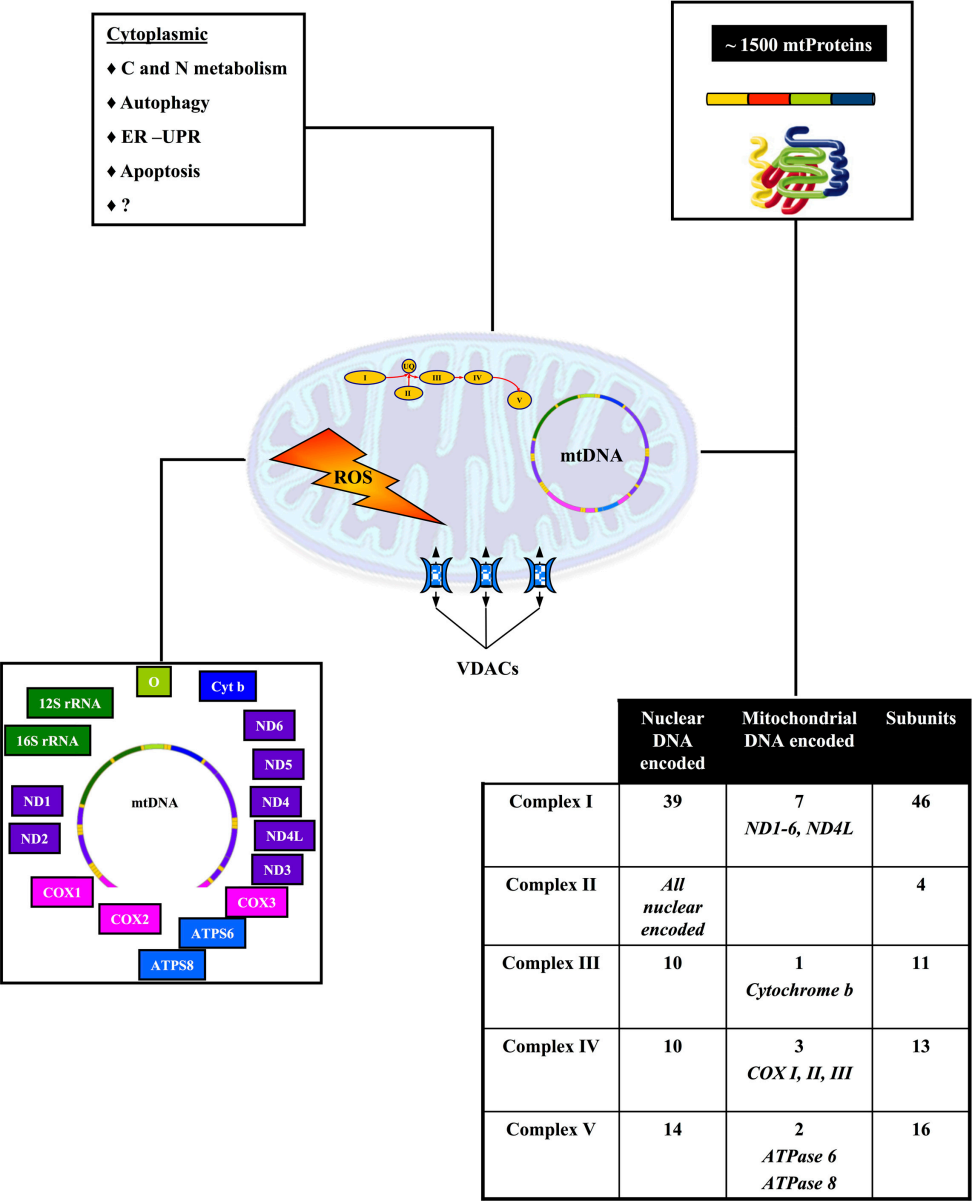
**Biogenesis of mitochondria results from the interaction of two genomes.** The chart outlines the role of the nuclear and mitochondrial genomes in mitochondrial biogenesis and function. See text for further details.

Mitochondria are semi-autonomous organelles that change in shape, size and association in different tissues or physio-pathological conditions. They derive from an ancestral endosymbiotic bacterium. Accordingly, mitochondria (Figure 2) are characterized by the interaction of two genomes: nuclear and mitochondrial. Mitochondrial DNA (mtDNA) is found in high copy number (around 10^3^) in all eukaryotic cells, both in conditions of homoplasmia or heteroplasmia. Since evolution resulted in loss of several bacterial pathways—and their encoding genes—modern mitochondria encode only a small number of genes. The 16.6 Kb human mtDNA encodes 2 ribosomal RNA required for assembling of the 70 S mitochondrial ribosomes and 22 tRNA, that is the minimal requirement for the autonomous protein synthesizing machinery of the mitochondria. Only 13 of the 85 proteins composing the OXPHOS complexes are encoded by human mtDNA. All other mitochondrial proteins are encoded by the nuclear genome (Gabaldon and Huynen, 2004). MitoMiner (http://mitominer.mrc-mbu.cam.ac.uk/v2.0-2012_01) lists 1164 and 1265 proteins in the human and mouse mitochondrial proteome, respectively.

Mitochondria play a key role in cellular bioenergetics, ion homeostasis, carbohydrate, and fatty acids metabolism and cell signaling. They are at the center of several inter-connected important metabolic pathways, which are further connected to other important cellular functions (Schatz, 2007) (Figure 2). Consequently any local mitochondria dysfunction can potentially have direct or indirect effects on intra or extra mitochondrial metabolic pathways, which could lead to very complex phenotypes.

### The mitochondrial proteome

General issues for mitochondrial proteomics do not differ from general proteomics (Yates et al., 2009). Analysis and definition of the mitochondrial proteome is not a trivial task because of extensive biological and technical variability (Da Cruz et al., 2005). As mitochondrial studies progress, the amount of information regarding the mitochondrial proteome increases in size and complexity. Several on-line databases dealing with the human mitochondrial proteome have been recently listed (Gianazza et al., 2011) and new ones are constantly appearing. By way of example, MitoMiner (Smith et al., 2012) covers studies from eleven species and as more and more data from normal and diseased tissues will become available, it may contribute to the study of mitochondrial (dys)function in mitochondrial-associated diseases such as cancer.

MS in combination with a variety of separation methods is the principal methodology for proteomics. Fractionation steps—based on organelle separation, and/or on physiochemical properties—prior to MS analysis are mandatory for the identification of low abundant proteins. Most methods for studying the mitochondrial proteome rely on purification of mitochondria from cellular homogenates, although methods that allow to study mitochondrial proteins without resorting on cell fractionation have been recently reviewed (Gianazza et al., 2011).

The definition of contaminants of the mitochondrial proteome is not straightforward. As pointed out by a recent study in *S. cerevisiae* (Ben-Menachem et al., 2011), dual targeting may greatly increase the ability of the mitochondria to respond dynamically to changing environmental conditions and should be taken into account in studies of mitochondrial proteome. Accordingly, rigorous quantitative analysis and careful data mining is required in studies of the mitochondrial proteome (Abadi et al., 2009).

Proteomics techniques in which proteome fractionation—i.e., separation at the protein level—precedes sample digestion and subsequent peptide-level separation and detection are referred to as bottom-up techniques and allow a high number of identifications.

Gel-based methods require staining, protein excision, in-gel digestion with trypsin and MS analysis. One-dimensional gels separate highly abundant proteins according to their apparent molecular weight (MW) and are suitable for hydrophobic proteins, such as membrane proteins (Lefort et al., 2009). In a 2D gel approach, after S–S bridge reduction, mitochondrial proteins are first separated according to their isoelectric point (pI) and then by MW. 2D gels offer good separation and quantization of proteins in the sample, allow to provide sample quality control (e.g., degradation) and to visualize PTMs that modify either pI (e.g., phosphorylation, left horizontal shift) or the MW (e.g., glycosylation, vertical up-shift). For semiquantitative studies 2D fluorescence difference in-gel electrophoresis (DIGE), in which two or more samples, including the internal standard, are labeled with different fluorescent dyes and separated on a 2D gel, is the most accurate technique. Changes in intensity of the dyes indicate whether a protein is up- or down-regulated (Gelfi and De Palma, 2012). Mitochondrial studies using a 2D gel approach are discussed in Distler et al. (2008).

To overcome limitations of 2D gels, techniques based on reversed phase liquid chromatography can be used (shotgun approach). Sample is digested in solution to peptides that are fractionated before being directly analyzed by MS. With the shotgun approach, protein is not lost with extraction from the gel, however, high abundant proteins are digested prior to MS, so these high abundant fragments can blunt many fractions.

The intensity of a peak detected in a single mass spectrum is directly proportional to the ion concentration. Differential labeling with *heavy* and *light* tags specifically designed to react with a particular amino acid (cysteine or lysine) of proteins extracted from two samples, induces a mass shift of the labeled peptides which will appear as a doublet on the mass spectrum, allowing the calculation of the peptide ratio and hence up- or down-regulation of the corresponding protein (Smolka et al., 2002; Schmidt et al., 2005).

In top-down proteomics intact proteins are directly analyzed by MS. This strategy offers potential access to the complete protein sequence and the ability to locate and characterize PTMs. This approach has not been achieved on a proteome scale owing to the lack of intact protein fractionation methods that are well integrated with tandem MS. Recently (Tran et al., 2011), an overall four-dimensional separation of whole protein molecules before ion fragmentation by tandem MS and protein identification, led to the definition of the human proteome with extremely high molecular detail. A similar strategy may be appropriate for precise and sensitive quantitative studies of the mitochondrial proteome.

Reverse phase protein microarrays (RPMA)—a high-throughput proteomic technique that allows the quantification of a given marker in minute amounts of protein from biological specimens (Mueller et al., 2010)—has been applied to quantification of proteins of energy metabolism in normal and tumor biopsies of colorectal cancer patients (Aldea et al., 2011). As the knowledge of the mitochondrial proteome increases it will be possible to extend this technique to include a wider coverage of mitochondrial proteins.

#### The mitochondrial proteome and cancer

As outlined above, defining the mitochondrial proteome could be essential to shed light on the connection between mitochondrial dysfunction and tumorigenesis, ultimately leading to discovery of new oncologic biomarkers and/or therapeutic targets (Bottoni et al., 2012). Without any attempt to be exhaustive, in this section we will report findings in cancer tissues and cell lines where either the mitochondrial proteome has been directly analyzed or information relevant for mitochondrial function has been obtained. Hu and collaborators (Hu et al., 2011) have recently presented an overview of cancer-related changes in mitochondrial proteomics to which the reader is referred for further information.

Mazzanti et al. (Mazzanti and Giulivi, 2006; Mazzanti et al., 2006) analyzed by differential proteomics the energy metabolism pathway of matched samples of normal and cancer tissues and found an unbalanced coordination between nuclear- and mitochondria-encoded mitochondrial proteins. This shift correlated with altered oxidative phosphorylation in cancer cells. An up-regulation of the mitochondrial apoptotic pathway was also observed.

Xu et al. (2010) conducted quantitative proteomic analyses on breast cell lines, including normal, primary tumor and metastatic tumor lines isolated from a single patient and found that proteins involved in metabolic processes were the most deregulated in both tumorigenesis and metastasis. Among the novel identified markers is the mitochondrial import inner membrane translocase subunit, TIMM17A that may play an oncogenic role in breast cancer. Eleven mitochondrial proteins whose abundance was increased at least two-fold in adriamycin-resistent MCF-7 human breast cancer cells as compared to the sensitive counterpart have been identified (Strong et al., 2006). Mitochondrial proteins differentially expressed in various stages of breast cancer progression have recently been reported (Chen et al., 2011).

A significant shift in the relative concentrations of nuclear-*vs* mitochondrial encoded cytocrome C Oxidase (COX) subunits has been reported during prostate cancer progression (Herrmann et al., 2003). A correlation has been found between the level of subunits of the COX complex in different stages of prostate cancer and prostate cancer-derived cell lines, two of which are matched normal and tumor lines derived form the same prostate gland (Krieg et al., 2004).

A recent study conducted on colorectal cancer using shotgun proteomics with stable isotope labeling and MS confirmed mitochondrial dysfunction (Kang et al., 2012). 2D-gel electrophoresis profiles of isolated mitochondria from neuroblastoma cells treated with sub-cytotoxic concentrations of a Complex I inhibitor, allowed to identify a variety of modulated mitochondrial proteins (Burte et al., 2011). Notably, changes in chaperones suggest a regulated link between Complex 1 inhibition and protein folding, while alterations in the levels of the multifunctional protein VDAC1 (see “Post-translational Modifications”) may be a signaling link between mitochondria and the rest of the cell prior to cell death.

#### Post-translational modifications

Another issue is presented by the identification of PTMs which play an essential role in cell signaling. Phosphorylation, S-nitrosylation (SNO), O-linked-β-N-acetylglucosamine glycosylation (O-GlcNAc), glutathiolation, sumoylation, ubiquitination are the most relevant PTMs of mitochondrial proteins. PTM MS-based proteomics is in most cases sensitive enough to identify low abundant proteins only after extensive upstream fractionation. Comparative techniques have been developed to cope with the differential abundance and the high sensitivity.

Lam et al., recently reported an adaptable, sensitive, specific and robust workflow for quantification of endogenous phosphopeptides from outer and inner mitochondrial membrane proteins (Lam et al., 2012). The development of a similar quantitative workflow for normal and cancer tissues of different histological origin could be instrumental in advancing knowledge and understanding of the regulatory effects of mitochondrial protein phosphorylation in cancer pathophysiology.

Binding of proteins to—and internalization into—mitochondria and post-translational modification of both mitochondrial and extramitochondrial proteins plays a key role in mitochondrial signaling. Voltage-dependent anion channels (VDAC1-3) are abundant β-barrel, channel-forming proteins located in the outer mitochondrial membrane, but also present in the plasma membrane (De Pinto et al., 2010). VDAC proteins are involved in ion and ADP/ATP exchange between the cytosol and the mitochondrion, as well as in the control of apoptosis—by acting as specific docking sites for a variety of proteins, including hexokinase and proapoptotic proteins (Shoshan-Barmatz and Golan, 2012). VDAC proteins are subjected to extensive PTMs, notably phosphorylation and acetylation, although little is known so far on the impact of these PTMs on VDAC pathophysiology.

Several reports have identified eleven, four and three phosphorylation sites in VDAC1, 2, and 3, respectively, as reviewed in Kerner et al. (2011), but only in a few cases a direct correlation between specific phosphorylations and VDAC activity has been reported. Phosphorylation of VDAC1 Ser^12,136^ sensitizes cells to apoptosis by extending the half-life of the phosphorylated VDAC1 form as compared to the non-phosphorylated one (Baines et al., 2007). A role for VDAC1 phosphorylation in prevention of cell death has been reported. Phosphorylation by Nek1 of VDAC1^S193^ protects cells against apoptosis, while ectopic expression of the non-phosphorylatable VDAC1^S193A^ and of the phosphomimetic VDAC1^S193E^ mutants resulted in cell death and apoptosis escape, respectively (Chen et al., 2009).

Two types of protein acetylations are known: N-terminal and lysine epsilon acetylation. A large fraction of proteins have been reported to be N-terminal acetylated (Arnesen et al., 2009; Zhang et al., 2011). The process is considered irreversible, but N-acetylation of some proteins is only partial (Goetze et al., 2009). N-acetylation may affect protein activity, stability, assembly, and intracellular location. Recently a biochemical assay that allows to correlate N-terminal acetylation with the availability of acetyl-CoA and the sensitivity to apoptotic stimuli has been developed (Yi et al., 2011). VDAC1, but not VDAC2, is N-terminal acetylated (Distler et al., 2007). The physiological role of this modification on VDAC function is presently unknown.

Increasing evidence suggests that reversible acetylation of mitochondrial proteins on lysine residues represents a key mechanism by which mitochondrial functions are adjusted to meet environmental demands (Lombard et al., 2011). The level of lysine acetylation of a given protein depends on the balance between the activity of protein acetyltransferases and deacetylating enzymes, i.e., histone deacetylases (HDAC) and sirtuins (NAD^+^-dependent deacetylases) (Zhao et al., 2010). No quantitative differences in acetylation for VDAC1 and VDAC2 were reported in fed *vs* calorie-restricted mice. While VDAC3^K28,63,109^ are acetylated constitutively, VDAC3^K20,61,226^ are differentially acetylated in livers of starved vs. those of fed mice (Kim et al., 2006). No direct correlation between specific acetylation events and VDAC activity has yet been reported. Combined proteomics/bioinformatics approaches that are being developed to study the mouse acetylome (Fritz et al., 2012) may prove valuable in understanding the role of this PTM in cancer biology.

### Mitochondrial bioenergetics

The Chemiosmotic Theory of oxidative phosphorylation states that the connection of electron transfer to ATP synthesis is indirect, via a H^+^ electrochemical gradient that is established by coupling electron flow through the four Complexes (I–IV) of the electron transport chain to proton extrusion from the mitochondrial matrix, 10 protons being ejected for each 2 electrons transferred from NADH to oxygen. Following the concentration gradient, protons return to the matrix via the Fo subunit of the FoF1 ATP synthase (Complex V). The proton flux induces conformational changes of ATP synthase, so to allow endoergonic ATP synthesis (Mitchell, 2011). Impairment of complexes I–IV disrupts electron flow and may cause mitochondrial respiratory dysfunction. Since, as outlined above, mitochondria may be central in the development of cancer, the study of the physio-pathological bioenergetic properties of mitochondria should prove instrumental in understanding cellular transformation.

Technologies are now available to monitor on a large scale several bionergetic-related activities: from oxygen consumption to ATP production, from fermentative activity to maximal respiratory capacity (Wu, 2009; Horan et al., 2012) and different papers describing their use in the study of cancer cell metabolism have been published (Wu et al., 2007b; de Groof et al., 2009; Chen and Shtivelman, 2010; Oliva et al., 2010; Pike et al., 2010; Guo et al., 2011; Vlashi et al., 2011; Fabian et al., 2012; Garcia-Cao et al., 2012; Sassi et al., 2012).

Hyperpolarization of the mitochondrial inner membrane has been detected in cancer cells. Since the rate of pyruvate oxidation in mitochondria in cancer cells is lower than in normal cells (Bonnet et al., 2007), such hyperpolarization is unlikely to be due to respiration. Consistently, increasing respiration of glycolytic cancer cells through inhibition of lactate dehydrogenase (Fantin et al., 2006; Le et al., 2010) or activation of PDH (Bonnet et al., 2007), relieves hyperpolarization. In a second redox process, molecular oxygen is converted to ROS, namely the superoxide anion radical (O^−^_2_) and H_2_O_2_. Mitochondria are selectively vulnerable to oxidative damage (Wallace, 1999). Mammalian Complex I (NADH:ubiquinone oxidoreductase) catalyzes the oxidation of NADH in the matrix and plays a major—and possibly unique—role in mitochondrial H_2_O_2_ production.

Much interest is currently given to the connection between ROS levels, mitochondrial function and autophagy that has been proposed as a major sensor of redox signaling (Lee et al., 2010). The relationship of autophagy with cancer is double-sided. Since autophagy-deficient mice are more likely to develop tumors (Marino et al., 2007; Mathew et al., 2009), autophagy apparently promotes survival of tumor cells and may contribute to resistance to chemotherapy. Down-regulating expression of essential autophagy proteins impairs growth of *ras*-dependent cancer cells (Guo et al., 2011). On the other hand, autophagic clearance of damaged proteins, organelles and DNA protects from tumorigenesis (Chen et al., 2009). Autophagy has also been shown to induce cell senescence, which is able to stop cancer progression (Young et al., 2009).

### Studying the dynamics of metabolic network: metabolic flux analysis with stable isotope tracers

Metabolomics generates detailed “snapshots” of biological processes (Hiller et al., 2009) by measuring metabolite concentrations, just as transcriptomics and proteomics generate snapshots of the level of transcripts and proteins. In order to dynamically study metabolic networks the fluxes of metabolites (i.e., *in vivo* reaction rates) need to be analyzed. Because cells and organisms fine tune their metabolism according to genetic, developmental and environmental clues, changes in flux result from interactions among proteins and metabolites, as well as from regulatory genetic and biochemical interactions, structural and allosteric enzyme regulation, etc., as reviewed in Sauer (2006).

The simplest way to measure a flux is to determine usage of a precursor and accumulation of the end-product. So for instance by assaying glucose consumption and lactate production, a rough estimate of the glycolytic flux can be obtained (Stephanopoulos, 1999). However such an analysis cannot give detailed information on intracellular fluxes and additional intracellular information must be obtained by isotope tracer experiments (Sauer, 2006; Le et al., 2012; Walther et al., 2012).

^13^C- (or ^15^N-) labeled substrates are fed to a growing cell population until the isotope label is distributed throughout the network, originating specific metabolite labeling patterns—detected through either MS or nuclear magnetic resonance (NMR)—according to flux distribution in the studied experimental situation. Obtaining fluxes from labeling data is not straightforward and requires computer model interpretation that rely on either parameter fitting procedures (Wiechert, 2001) or direct and local interpretation of selected labeling patterns, for example, the mass distribution of pyruvate (Fischer and Sauer, 2003). Computational evaluation of the use of ^13^C labeled glucose and glutamine and indications to direct choice of isotope tracer for tracking different pathways have been described (Metallo et al., 2009). Methodologies for non-targeted analysis of stable-isotope labeled metabolomics data are also available (Hiller et al., 2011).

Combining the use of isotopic tracers and computational algorithms, MFA enables quantitative estimation of intracellular fluxes allowing to describe the actual functionality of a given enzyme or pathway. The investigation of a metabolic pathway by MFA is the more direct way to derive the metabolic circuit (see section “From “Omics” Data to Networks and Beyond”) in any given condition allowing to detect non-canonical pathways. For instance in microorganisms it allowed to detect the unexpected activity of the Entner–Doudoroff pathway (Fuhrer et al., 2005) in *Actinomycetes*. In mammalian cells the non-canonical labeling of TCA cycle-associated metabolites has been demonstrated in transformed cell lines by MFA, that highlighted K-*ras*-induced decoupling of glucose and glutamine utilization (Gaglio et al., 2011).

MFA provides important data to extend understanding of flux regulation, for instance through techniques such as metabolic control analysis (MCA) (Fell and Black, 1997), and its extension aiming to quantitatively dissect purely metabolic from hierarchical (i.e., dominated by regulation of gene expression) regulation (ter Kuile and Westerhoff, 2001). MFA also provides reference data sets that can be used to check predictions of mathematical models or as input data for constraining parameter estimation, the ultimate goal being to integrate all relevant experimental data in order to quantitatively explain and predict metabolic regulation and cellular phenotypes.

### Bioimaging: PET and MR techniques for the study of cancer metabolism *In vivo*

Application to cancer of *in vivo* imaging procedures like Positron Emission Tomography (PET) and Magnetic Resonance (MR) is increasing. Since PET allows to visualize modifications in cell metabolism or tissue microenvironment, it represents a unique tool for a better understanding of cancer biology, the set up of novel diagnostic procedures and for early assessment of the efficacy of target-directed therapies.

In recent years a growing number of radiopharmaceuticals have been developed and validated at both preclinical and clinical levels for the management of patients with different cancers. Different tracers allow to visualize and measure hallmark cancer phenotypes including glucose and fatty acid metabolism, cell proliferation or regional hypoxia.

Uptake of the glucose analog 2-deoxy-2-(^18^F)-D-glucose (FDG) is widely used in clinical practice for staging, restaging and early prediction and assessment of response to pharmacological treatment. The lack of the hydroxyl group in position 2 blocks FDG metabolism past GLUT-mediated glucose transport and phosphorylation by hexokinases (HK): hence FDG uptake specifically reflects the levels of GLUT and HK activity (Haberkorn et al., 2011). The interest in FDG as marker of drug efficacy rises from the observation that glucose metabolism and in particular GLUT and HK levels are controlled by the same pathways where most of the novel targeted therapy act (Honer et al., 2010) and modifications in FDG uptake represents an early event in case of response to treatment with tyrosine kinase receptors agents like Imatinib or Erlotinib, or mTOR inhibitors like Everolimus (Cullinane et al., 2005; Shankar et al., 2006; Sunaga et al., 2008; Aukema et al., 2010).

Many efforts have been recently dedicated to developing PET radiopharmaceuticals for *in vivo* imaging of glutamine metabolism that is now recognized to play a central role in the metabolism of proliferating cells (see section “Mitochondrial Metabolism Remodeling in Cancer Cells”). The potential use of 3′-deoxy-3′-[^18^F]*fluorothymidine* (FLT)—whose uptake reflects the activity of cytosolic thymidine kinase-1 (TK-1) which is the first enzyme in the salvage pathway of DNA synthesis activated during the S-phase of the cell cycle (Soloviev et al., 2012)—as a biomarker for therapy efficacy is under evaluation at both clinical and preclinical levels. FLT allows indirect monitoring of modifications in metabolic pathways including glutaminolysis, affecting cancer cell phenotype by evaluating its effects on tumor growth.

More recently, two tracers of potential interest for *in vivo* imaging of glutamine metabolism have been described and validated in preclinical models of cancer: ^18^F-(2S, 4R)4-fluoroglutamine and l-[5-^11^C]-glutamine (Lieberman et al., 2011; Qu et al., 2012). These tracers are taken up and specifically retained by cancer cells giving adequate signal to noise ratios. However further preclinical studies are needed to better demonstrate if their accumulation fully reflects glutaminolysis (Lieberman et al., 2011; Qu et al., 2012). *In vivo* imaging of glutamine metabolism is of great relevance, since it could be used alone for low glycolitic tumors which might use glutamine as an alternative nutrient, or—in combination with FDG—to identify tumors in which persistent glutamine metabolism will support cell survival and negatively affect the outcome as well as to explore possible links with specific mutations (Rajagopalan and DeBerardinis, 2011).

Another emerging technique for the *in vivo* measurement of cancer metabolism and glutamine utilization is Magnetic Resonance Spectroscopy (MRS) that allows quantification of different tissue metabolites including glutamine and glutamate. However, MRS indicates only the global levels of metabolites inside tissue and not their relative fluxes between intracellular and extracellular spaces. This measurement is possible and traditionally performed *in vitro* using isotopically labeled compounds. In the field of MR, hyperpolarized ^13^C MR represents an emerging tool for the real-time monitoring of single step reactions along metabolic pathways. In hyperpolarized MR, a molecule is labeled with an NMR-active nucleus and then hyperpolarized using dynamic nuclear polarization. Nuclear spin hyperpolarization can dramatically increase the sensitivity of ^13^C MRS, allowing dynamic measurements of the metabolism of hyperpolarized ^13^C-labeled substrates *in vivo*. To date, [5-^13^C]-glutamine, [1-^13^C]-glutamate, and [5-^13^C-4-^2^H2]-glutamine have been successfully used in cells, but their implementation *in vivo* at preclinical stage remains limited. Polarization tends to decay relatively rapidly following injection into living tissues minimizing the time frame during which the signal can be detected. Strategies allowing improvements in polarization levels of these agents might pave the way for wider *in vivo* implementation (Sibson et al., 1997; Gallagher et al., 2008, 2011; Qu et al., 2011).

### Modeling mitochondrial activities

Models describing mitochondrial bioenergetics vary in scope, complexity and in their applicability to different systems. Thermodynamic models were the first to be utilized, but more recently, kinetic or mixed thermodynamic-kinetic models were introduced, as reviewed in Cortassa and Aon (2012). In the simplest approach, the respiration flux through the whole mitochondria is described by a single empirical oxygen consumption equation. Recently, a modular kinetic rate equation—the chemiosmotic rate law—expressing the mitochondrial flux has been proposed (Chang et al., 2011). It describes mitochondrial flux through three configurable modulating factors. By allowing selective configuration of the system and selection of its kinetic properties, such an approach may allow comparative analysis of mitochondria in different physio-pathological states.

Different models in which respiration is analyzed with reference to the molecular component involved, have been proposed (Korzeniewski and Zoladz, 2001; Yugi and Tomita, 2004; Wu et al., 2007a). In particular, the Beard group described a computational model of mitochondrial metabolism of human muscle cells including the Tricarboxylic Acid Cycle, oxidative phosphorylation, metabolite transport and electrophysiology (Wu et al., 2007a). The model, constructed on the basis of detailed kinetics and thermodynamically balanced reaction mechanisms has been validated by its application to diverse conditions, including *in vitro* data on isolated mitochondria and *in vivo* experimental measurements. The same group has developed a database of thermodynamic properties that includes glycolysis, tricarboxylic acid cycle and reactions of the pentose phosphate pathway (Li et al., 2011).

Mitochondrial metabolism has also been studied according to the Flux Balance Analysis (FBA) paradigm. In a first study, FBA has been used to characterize the optimal flux distributions for maximal ATP production in mitochondria with the aim to offer a systemic perspective regarding the effect of stoichiometric constraints and specific metabolic fluxes on mitochondrial function (Ramakrishna et al., 2001). A refined, manually curated FBA metabolic model of the mitochondrion has been recently published (Smith and Robinson, 2011) that builds extensively on the MitoMiner mitochondrial protein database (Smith et al., 2012) and has been used to calculate metabolite fluxes under normal and pathological conditions, including deficiencies in fumarase, succinate dehydrogenase and AKG dehydrogenase.

None of the mentioned reports explicitly connects mitochondrial metabolism to signaling pathways that may impinge on mitochondria functionality, nor to signaling pathways that take place within the mitochondria. A first step in this direction is found in a recent paper (Huber et al., 2011) describing a computational systems biology study that integrates mitochondrial bioenergetics and apoptotic signaling. Simulation results suggest that enhanced glucose utilization of cancer cells may counteract the lethal bioenergetic crisis that would stimulate apoptosis. Since the metabolic and signaling role of mitochondria are strongly interconnected, it is expected that in a near future modeling efforts aiming to connect oncogenic signaling and metabolism should appear and contribute to understanding of the function of the mitochondria in cancer.

Most modeling efforts aim to describe mitochondrial bioenergetics properties with a given level of granularity, as deemed appropriate for intended purpose of the model. Sometimes even the same authors present models of the same system with different resolution (Klipp et al., 2005; Gennemark et al., 2006). Multiscale modeling is indeed more a necessity than a nuisance (Noble, 2002; Kitano, 2010). To be effective, modeling efforts need to be put in place within a general framework that allows to highlight input/output and regulatory connections among different modules.

A recent study (Cloutier and Wellstead, 2010) presented a generic energy metabolism model which describes energy metabolism in terms of engineering control mechanisms and structures. Following their interest in Parkinson's disease, the same authors proposed (Wellstead and Cloutier, 2011) to use a mathematical model of brain energy metabolism (Cloutier et al., 2009) as the core module to which connect other modules describing processes associated with the disease. Such a modular model can act as a scaffold for modules of different molecular granularity and may also allow easy reshaping of structural and regulatory connections as new data become available.

In the next section we discuss our roadmap for the development of a structured, system-level model of the *enhanced growth* property of cancer cells, in which remodeling of mitochondrial metabolism is going to have a major role. Full understanding of this property is going to be crucial for biological understanding and in perspective, for personalized treatment of cancer.

## Conclusions and perspectives

Taking together the findings previously discussed, it is possible to state that two properties of the phenotype of K-*ras* transformed cells, i.e., “enhanced proliferation” and “LG-induced cell death,” are closely linked. They are shown to depend upon a metabolic remodeling given by enhancement of glycolysis and by rewiring of mitochondrial metabolism, with down-regulation of TCA cycle activity, increase of reductive carboxylation of AKG, derived from glutamine, followed by sustained production of building blocks and glutathione.

This substantial change in cellular metabolism can be connected, in a causal relationship, to other molecular events characteristic of K-*ras* transformed cells (Figure 1). In fact, the activation of signaling pathway by oncogenic K-*ras* is able to strongly activate the uptake of glucose and of glutamine (Levine and Puzio-Kuter, 2010; Gaglio et al., 2011). The increased flux of glucose and the concomitant activation by the PI3K and PKB/AKT pathways—downstream of K-*ras*—of several key glycolytic enzymes are able to increase the glycolytic flux (Gaglio et al., 2009, 2011). It is interesting to recall that systemic elevation of PTEN, the main negative regulator of PI3K signaling, is able to reduce glucose and glutamine uptake, increase mitochondrial oxidative phosphorylation, reduce glutaminolysis and lactate production, making cells resistant to oncogenic transformation (Garcia-Cao et al., 2012). In the meantime the activation of K-*ras* down-regulates the cAMP/PKA pathway and this event impacts on mitochondrial Complex I assembly/function, strongly reducing its activity (Palorini et al., 2012). Thus, pyruvate generated by the stimulated glycolysis may find obstructed the way to the TCA cycle. This condition may be enhanced by both *MYC* activation and hypoxia, that are able to activate pyruvate dehydrogenase kinase-1 (PDK1), which inactivates PDH converting pyruvate to Acetyl-CoA (Bonnet et al., 2007), the resulting overflow of pyruvate being directed towards the production of lactate.

The stimulated uptake of glutamine and the concomitant activation of glutaminase and glutamine dehydrogenase lead to AKG production. Mitochondrial IDH2 converts AKG to citrate by reductive carboxylation, that can be stimulated by NADH, likely building up due to Complex I inactivation. The high level of NADH, through nicotinamide nucleotide transhydrogenase (NNT) will produce NADPH at the expenses of NADP^+^ to stimulate reductive carboxylation of AKG to citrate. Citrate, exported into the cytoplasm, is able to sustain “*de novo*” synthesis of fatty acids, a typical cancer growth feature (Metallo et al., 2011; Wise et al., 2011; Icard et al., 2012). Production of aspartate from oxalocetate, catalyzed by aspartate amino transferase, opens the way to the production of building blocks, such as amino acids, nucleotides, glutathione. Contrary to the behavior observed in normal cells, that utilize glutamine mostly as a nitrogen source, exporting glutamate into the medium, in cancer cells glutamine serves as source of both carbon and nitrogen (Gaglio et al., 2011) and plays a role in quenching ROS produced by defective Complex I (Gaglio et al., 2011).

From this simple and so far still incomplete analysis, it is possible to justify many aspects of the “enhanced growth” phenotype and show that this function/system-level property is sustained by a large number of biochemical pathways that interact often in ways that are not coincident with canonical indications. At first glance this behavior recalls that of a “Rube Goldberg machine,” which performs a given task in a very complex fashion, interlocking in an unexpected way elementary sequences of events that realize a chain reaction to perform the required task. The interest of the chain reaction is given by the fact that the first reaction generates a product that determines the behavior of the second reaction and so on. For instance, when glucose uptake and glycolytic flux are low, pyruvate is utilized by the TCA cycle and sustains OXPHOS-dependent ATP synthesis. Instead when the glycolytic flux strongly increases, pyruvate is mostly diverted to lactate production. Therefore the energy released at the various reaction steps allows the system to reach one or the other end point, as a function of the conditions generated during the chain.

**Figure 3:**
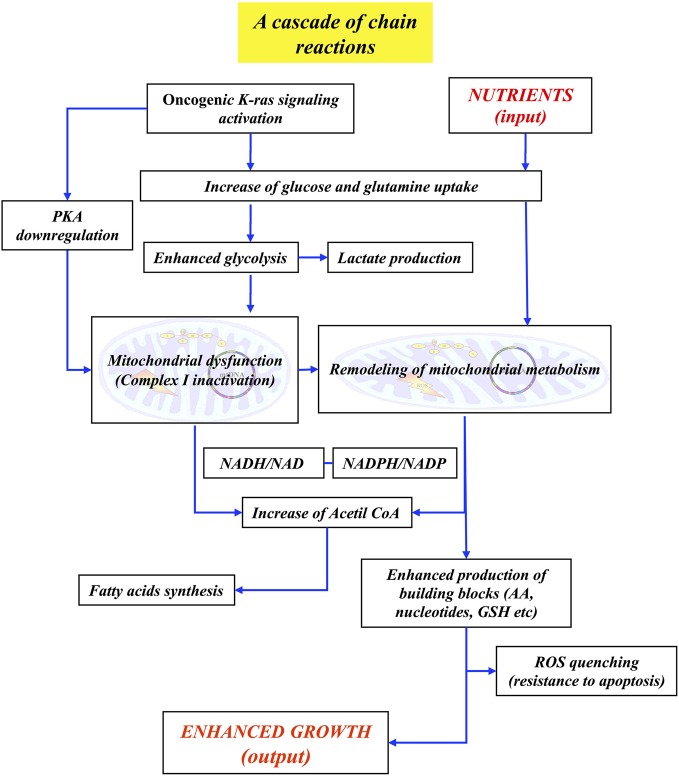
**Low resolution concept map of the “enhanced growth” property in cancer cells.** The major events—and their interconnections—leading to the enhanced growth phenotype are presented. See the text for details.

Of course Figure 3 represents only a first attempt to construct, as indicated previously in section “From “Omics” Data to Networks and Beyond,” a concept map of molecular events that underlay the “enhanced growth” function of K-*ras* transformed cells. The first step to be undertaken afterwards is to verify its completeness and eventually to add other relevant pathways, not yet considered. Then, efforts of constructing mathematical models, at the appropriate level of detail, may be undertaken.

As discussed in section “Modeling Mitochondrial Activities,” models of mitochondrial functions are quite complex and their granularity depends upon the problems one would like to address. A first interesting question to investigate could be the role of the altered NADH/NAD^+^ ratio, due to Complex I partial inactivation, on the insurgence and maintenance of the mitochondrial remodeling of the glutamine utilization in cancer cells.

A neat definition, at molecular level, of the structure of the complex molecular machine underlying “enhanced growth,” compared to those of both growing and resting normal cells could allow to identify the step(s) to be inhibited, if one wants to specifically arrest growth of cancer cells, leaving unaffected normal ones.

Following the same line of thought, Figure 3 could be the basis for a concept map that aims to identify the relevant molecular pathways involved in the “LG-induced cell death.” Of course this second concept map needs to be largely implemented: first of all one should know which are the metabolic reactions that take place, at limiting glucose, in K-*ras* transformed cells as compared with *myc*-dependent Burkitt lymphoma cell lines which instead are able to survive using only glutamine metabolism (Le et al., 2012). Besides, a careful analysis and unambiguous molecular definition (Galluzzi et al., 2012) of the pathway(s) that brings to cell death K-*ras* transformed cells in low glucose will be required.

Putting together this information, one should be able to construct first a concept map, then a mathematical model of “LG-induced cell death.” Based on this model, extensive *in silico* simulations can be carried on, and their predictions compared and validated with experimental results and data sets. As noted above, multiscale modeling and simulation tools play an essential role. The identification of the molecular steps that, inhibited by a new drug, may be able to selectively kill K-*ras* dependent cancer cells, offers hope to become able to eradicate this type of K-*ras*-addicted cancers.

In conclusion, as discussed in this paper, post-genomic analysis of cancer cells becomes able to yield understanding and predictive ability for understanding of multifactorial diseases and hence develop efficient new therapeutic treatments, only by integration of network analysis with dynamic modeling of physiologically relevant system-level properties, thus modifying the initial, too optimistic, expectation (Henney and Superti-Furga, 2008) which proposed that network analysis alone could be sufficient.

### Conflict of interest statement

The authors declare that the research was conducted in the absence of any commercial or financial relationships that could be construed as a potential conflict of interest.
